# Deep imaging of LepR^+^ stromal cells in optically cleared murine bone hemisections

**DOI:** 10.1038/s41413-024-00387-9

**Published:** 2025-01-13

**Authors:** Yuehan Ni, Jiamiao Wu, Fengqi Liu, Yating Yi, Xiangjiao Meng, Xiang Gao, Luyi Xiao, Weiwei Zhou, Zexi Chen, Peng Chu, Dan Xing, Ye Yuan, Donghui Ding, Ge Shen, Min Yang, Ronjie Wu, Ling Wang, Luiza Martins Nascentes Melo, Sien Lin, Xiaoguang Cheng, Gang Li, Alpaslan Tasdogan, Jessalyn M. Ubellacker, Hu Zhao, Shentong Fang, Bo Shen

**Affiliations:** 1 College of Life Sciences, Beijing Normal University, 100875 Beijing, China; 2 National Institute of Biological Sciences, Beijing (NIBS), 102206 Beijing, China; 3 Peking University-Tsinghua University-National Institute of Biological Sciences Joint Graduate Program, School of Life Sciences, Tsinghua University, 100084 Beijing, China; 4 School of Biopharmacy, China Pharmaceutical University, 211198 Nanjing, China; 5 Chinese Institute for Brain Research, Beijing (CIBR), 102206 Beijing, China; 6 Chinese Academy of Medical Sciences & Peking Union Medical College, 100730 Beijing, China; 7 Peking University-Tsinghua University-National Institute of Biological Sciences Joint Graduate Program, Academy for Advanced Interdisciplinary Studies, Peking University, 100871 Beijing, China; 8 Arthritis Clinic and Research Center, Peking University People’s Hospital, Peking University, 100044 Beijing, China; 9 Musculoskeletal Research Laboratory, Department of Orthopaedics & Traumatology & Li Ka Shing Institute of Health Sciences, Faculty of Medicine, The Chinese University of Hong Kong, 999077 Shatin, Hong Kong SAR PR China; 10 Department of Radiology, Beijing Jishuitan Hospital, Capital Medical University, National Center for Orthopaedics, 100035 Beijing, China; 11 Department of Dermatology, University Hospital Essen & German Cancer Consortium, Partner Site, Essen, 45147 Germany; 12 Department of Molecular Metabolism, Harvard T.H. Chan School of Public Health, Boston, MA 02115 USA; 13 Tsinghua Institute of Multidisciplinary Biomedical Research, Tsinghua University, 100084 Beijing, China

**Keywords:** Bone, Bone quality and biomechanics

## Abstract

Tissue clearing combined with high-resolution confocal imaging is a cutting-edge approach for dissecting the three-dimensional (3D) architecture of tissues and deciphering cellular spatial interactions under physiological and pathological conditions. Deciphering the spatial interaction of leptin receptor-expressing (LepR^+^) stromal cells with other compartments in the bone marrow is crucial for a deeper understanding of the stem cell niche and the skeletal tissue. In this study, we introduce an optimized protocol for the 3D analysis of skeletal tissues, enabling the visualization of hematopoietic and stromal cells, especially LepR^+^ stromal cells, within optically cleared bone hemisections. Our method preserves the 3D tissue architecture and is extendable to other hematopoietic sites such as calvaria and vertebrae. The protocol entails tissue fixation, decalcification, and cryosectioning to reveal the marrow cavity. Completed within approximately 12 days, this process yields highly transparent tissues that maintain genetically encoded or antibody-stained fluorescent signals. The bone hemisections are compatible with diverse antibody labeling strategies. Confocal microscopy of these transparent samples allows for qualitative and quantitative image analysis using Aivia or Bitplane Imaris software, assessing a spectrum of parameters. With proper storage, the fluorescent signal in the stained and cleared bone hemisections remains intact for at least 2–3 months. This protocol is robust, straightforward to implement, and highly reproducible, offering a valuable tool for tissue architecture and cellular interaction studies.

## Introduction

Adult bone marrow leptin receptor-expressing stromal cells (LepR^+^ cells) secrete key growth factors to maintain hematopoietic stem/progenitor cells,^1–10^ whereas they themselves give rise to bone and fat.^11–13^ Recent single-cell RNA-sequencing studies suggest that LepR^+^ cells are heterogeneous especially under stress and include skeletal stem cells, osteogenic progenitors, adipogenic progenitors, fibroblasts, etc.^13–24^ Unraveling the spatial interaction of LepR^+^ stromal cells with other compartments in the bone marrow, such as the endothelium or the hematopoietic stem/progenitor cells, will shed light on our understanding on the skeletal tissue. Currently, however, there are few markers available to separate these subpopulations in situ and lack of a reliable monoclonal antibody for these cells, thus hindering further investigations of their specific functions.^25–28^

To identify the locations of these subpopulations of LepR^+^ cells, we developed a tissue clearing protocol that enables immunofluorescence visualization of mouse bone marrow in three dimensions by making the tissue transparent.^4,9,29^ In this protocol, we described how to prepare opaque organs and tissues of interest (here, we use mostly mouse long bones as examples), stain the tissue with fluorescence-conjugated antibodies, and obtain the distribution pattern of distinct cell populations (here, mostly LepR^+^ cells, endothelial cells, or their subsets) in three dimensions after optical clearance. We previously have used this approach to demonstrate that the Osteolectin-expressing subset of LepR^+^ cells (Osteolectin^+^ cells) resides exclusively in a peri-arteriolar location^4^ and that nerve growth factor (NGF) is primarily synthesized by the perivascular LepR^+^ cells.^9^ Here, we now modified this approach to be more cost-efficient and less time-consuming to image various stromal cell populations in bone marrow and further adapted this protocol to investigate other cell populations in bone marrow or other perivascular or vascular cells across diverse mammalian tissues, such as the spleen and lung.

## Results

### Overview of the modified workflow for the deep imaging technique

**Fig. 1:**
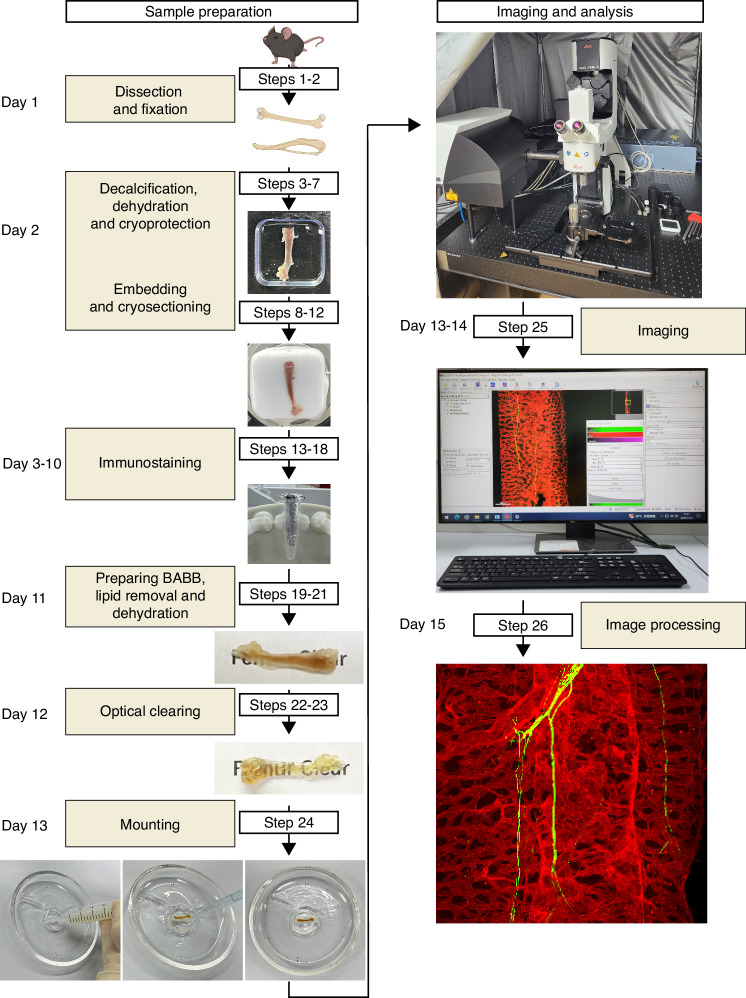
Workflow of the optimized deep imaging protocol for optically cleared skeletal tissue. Steps for preparing the deep-imaging bone hemisections, including tissue fixation, decalcification, dehydration, embedding, and cryosectioning to generate bone hemisections; and immunostaining, tissue clearing, and mounting, with subsequent imaging. BABB, benzyl alcohol/benzyl benzoate (1:2, v/v) clearing solution

High-resolution visualization of rare cell populations throughout an entire organ can enhance our understanding of their spatial distribution, interactions, and functions. We developed a deep imaging method to visualize LepR^+^ cells and hematopoietic stem/progenitor cells in bone marrow from the long bones of adult genetic reporter mice primarily with antibodies targeting tdTomato (tandem dimer Tomato) and GFP (green fluorescent protein).^4,29^ In this article, we detail a method for optically clearing tissues post-immunofluorescence staining. The optimized process is outlined in a flowchart (Fig. 1) and a detailed procedure (Table 1). We have refined this method specifically for long bones from the mouse skeletal system and validated it with various antibodies, as listed in Table 2.

**Table 1:** Procedure for deep imaging optically cleared mouse bone hemisections

Step	Procedure
**Sample preparation (timing: 1** **h)**	
1	Dissect femurs and tibias by carefully removing all surrounding tissues.
**Fixation, decalcification, dehydration, and cryoprotection (timing: 1.5 days)**	
2	Fix the bones with ice-cold 4% PFA for 15 min and incubate at 4 °C on a rotator for 6 h.
3	Wash the bones twice with PBS.
4	Incubate the bones with EDTA under constant agitation on a rotator at 4 °C for 12 h.
5	Wash the bones twice with PBS.
6	Incubate the bones in ice-cold 30% sucrose solution under constant agitation on a rotator at 4°C for 12 h.
7	Remove the sucrose solution from the bones and proceed to OCT embedding.
**Preparing hemisections (timing: 2** **h)**	
8	Embed the bones by carefully placing it in OCT.
9	Freeze the samples on flat dry-ice to allow the OCT to solidify completely.
10	Place the frozen tissue mold in a pre-cooled (–20 °C) cryostat for 30 min–1 h before sectioning.
11	Glue the block to the holder using OCT at –20 °C.
12	Cut the long bones until the sinus and the full length of the long bone are fully exposed.
**Immunostaining (timing: 8 days)**	
13	Transfer the hemisections to 1.5-mL microcentrifuge tubes, place them on ice, respectively. Wash the hemisections twice with 1.5 mL of PBS to remove the OCT completely.
14	Discard the PBS and block the hemisections with 5% donkey serum in 0.5% PBST for 6 h at room temperature with shaking.
15	Transfer the hemisections to 0.6-mL microcentrifuge tubes with freshly prepared primary antibody solution respectively and incubate at room temperature with shaking for 3 days.
16	Transfer the hemisections to new 1.5-mL centrifuge tubes respectively and wash with PBS three times for 15 min each at room temperature. Then wash with PBS with shaking at room temperature overnight.
17	Transfer the hemisections to 0.6-mL microcentrifuge tubes with freshly prepared secondary antibody solution respectively and incubate at room temperature with shaking for 3 days in the dark.
18	Perform PBS washes as in Step 16.
**Optical clearing (timing: 2 days)**	
19	One day before optical clearing, prepare the BABB buffer. Incubate the mixture with shaking for at least 24 h.
20	Spin down the BABB mixture at 800×*g* for 15 min at room temperature.
21	Dehydrate hemisections with at least three 5-minute washes with 1.5 mL methanol.
22	Remove methanol buffer from the tube and replace with 1.5 mL BABB. Incubate the hemisections in BABB with shaking at room temperature in the dark for several hours to overnight. Repeat this step if necessary with fresh BABB for optimal clearing.
23	Remove the used BABB and fill the microcentrifuge tube with fresh BABB. Store the hemisections at 4°C in BABB in the dark for up to at least 3 months.
**Preparing mounting dish (timing: 1** **h)**	
24	Squeeze a small amount of silicone onto the center of quartz mold. Place the hemisection on top of the fresh silicone. Make sure the hemisection is oriented with the marrow cavity facing up. Add a few drops of BABB to the hemisection and silicone. Let the silicone foundation solidify at room temperature for 30 min before filling the mold with fresh BABB to immerse the hemisection.
**Image acquisition and processing (timing: 2 days)**	
25	Use a resonant confocal laser-scanning microscope with *z*-stack scanning and tiling (e.g., Leica Stellaris or Leica SP8) to obtain sequential depth images.
26	Convert the tiled z-stack images into “.ims” or “.aivia.tif” format using Imaris x64 10.1.0 (Bitplane) or Aivia 12.1.0 respectively, followed by three-dimensional rendering, snapshot generation, and quantification.

**Table 2:** Antibodies successfully used with this method

Antibody	Recommended working concentration	Company	Cat. no.
**Primary antibodies**			
Chicken anti-green fluorescent protein	40 μg/mL	Aves Labs	GFP-1020
Rabbit anti-laminin Ab-1	1:200	Thermo Fisher Scientific	RB-082-A0
Goat anti‑discosoma tdTomato	12 ng/μL	LifeSpan Biosciences	LS-C340696
Rat anti-endomucin (V.7C7)	1:100	Santa Cruz	sc-65495
Goat anti-endomucin	1 ng/μL	R&D Systems	AF4666
Goat anti-VEGFR3/Flt-4	1:100	R&D Systems	AF743
Goat anti-VE-Cadherin	1:100	R&D Systems	AF1002
Rabbit anti-DsRed	1:250	Takara Bio	632496
Rabbit anti-laminin 1 + 2	2 ng/μL	Abcam	ab7463
Rabbit anti-peripherin	1:250	Abcam	ab4666
Biotin goat anti-CD117/c-Kit	1:250	R&D Systems	BAF1356
Rat anti-CD31/PECAM-1 (MEC 13.3)	1:100	Santa Cruz	sc-18916
Rabbit anti-Leptin Receptor	1:100	Abcam	ab318272
Biotin goat anti-Leptin Receptor	2 ng/μL	R&D Systems	BAF497
Biotin hamster anti-CD3e	1:100	BD Biosciences	51-01082 J
Biotin rat anti-CD11b	1:100	BD Biosciences	51-01712 J
Biotin rat anti-CD45R	1:100	BD Biosciences	51-01122 J
Biotin rat anti-Ly-6G and Ly-6C	1:100	BD Biosciences	51-01212 J
Biotin rat anti-Ter119/Erythroid cells	1:100	BD Biosciences	51-09082 J
FITC mouse anti-Actin, α-smooth muscle	1:250	Sigma-Aldrich	F3777
APC rat anti-Ly-6A/E (Sca-1)	2.5 ng/μL	Biolegend	122518
Rabbit anti-tRFP antibody	12 ng/μL	Evrogen	AB233
**Secondary antibodies**			
Donkey anti-goat IgG (H + L) cross-adsorbed secondary antibody, Alexa Fluor 555	4 ng/μL	Thermo Fisher Scientific	A21432
Alexa Fluor 488 AffiniPure F(ab’)2 fragment, donkey anti-rabbit IgG (H + L)	1.2 ng/μL	Jackson ImmunoResearch	711-546-152
Alexa Fluor 647 AffiniPure F(ab’)2 fragment, donkey anti-rabbit IgG (H + L)	1.2 ng/μL	Jackson ImmunoResearch	711-606-152
Alexa Fluor 488 AffiniPure F(ab’)2 fragment, donkey anti-chicken IgY (IgG) (H + L)	1.2 ng/μL	Jackson ImmunoResearch	703-546-155
Alexa Fluor 647 AffiniPure donkey anti-goat IgG (H + L)	4 ng/μL	Jackson ImmunoResearch	705-605-147
Donkey anti-rabbit IgG (H + L) highly cross-adsorbed secondary antibody, Alexa Fluor 546	1:250	Thermo Fisher Scientific	A10040
APC Streptavidin	1:200	Biolegend	405207
Alexa Fluor 594 AffiniPure Fab fragment donkey anti-rat IgG (H + L)	1:500	Jackson ImmunoResearch	712-587-003

In this study, we improved our blocking cocktail for cost-effectiveness and introduced an innovative, reusable platform for mounting optically cleared bone hemisections (Fig. S1). We reduced the blocking time, streamlining the sample preparation for imaging to 11–12 days without compromising antibody binding specificity. The reusable quartz mold for bone hemisections is versatile for other tissues, ensuring better preservation of stained and cleared samples post-imaging. The protocol yields multicolor immunofluorescent images from tiled scan-stacks, at least 500 µm deep, which can be reconstructed into 3D and projected into 2D at various magnifications and depths (Fig. S2a). This approach leverages multiple transgenic mouse models to locate rare cell populations, including stem/progenitor cells in skeletal tissue, at high-resolution with detailed spatial interactions (Fig. S2b).

### Method application in the long bone

Growth factors play vital roles in bone homeostasis, regeneration, and remodeling. Osteolectin, a novel bone growth factor, stimulates the osteogenic differentiation of bone marrow LepR^+^ cells and other progenitors into osteoblasts by activating integrin α11β1 and Wnt signaling.^30–33^ Using our optimized imaging technique, we confirmed that *Osteolectin*-mTomato^+^ (*Oln*-mTomato^+^) cells co-localized with LepR^+^ cells around endomucin^low^ arterioles in the diaphysis (Fig. S3a). Further staining of a femur hemisection from an *Oln*^*mTomato/+*^ mouse with endomucin (a vascular endothelial cell marker) and SCA-1 (a bone marrow arteriolar endothelial cell marker) revealed that *Oln*-mTomato^+^ cells were specifically associated with SCA-1^+^endomucin^low^ arterioles, not with sinusoids (Fig. S3b, c). Our enhanced deep imaging method has thus facilitated the identification of peri-arteriolar Osteolectin^+^ cells as a rare, short-lived osteogenic progenitor population, which were reported to be mechanosensitive.^4,34^

**Fig. 2:**
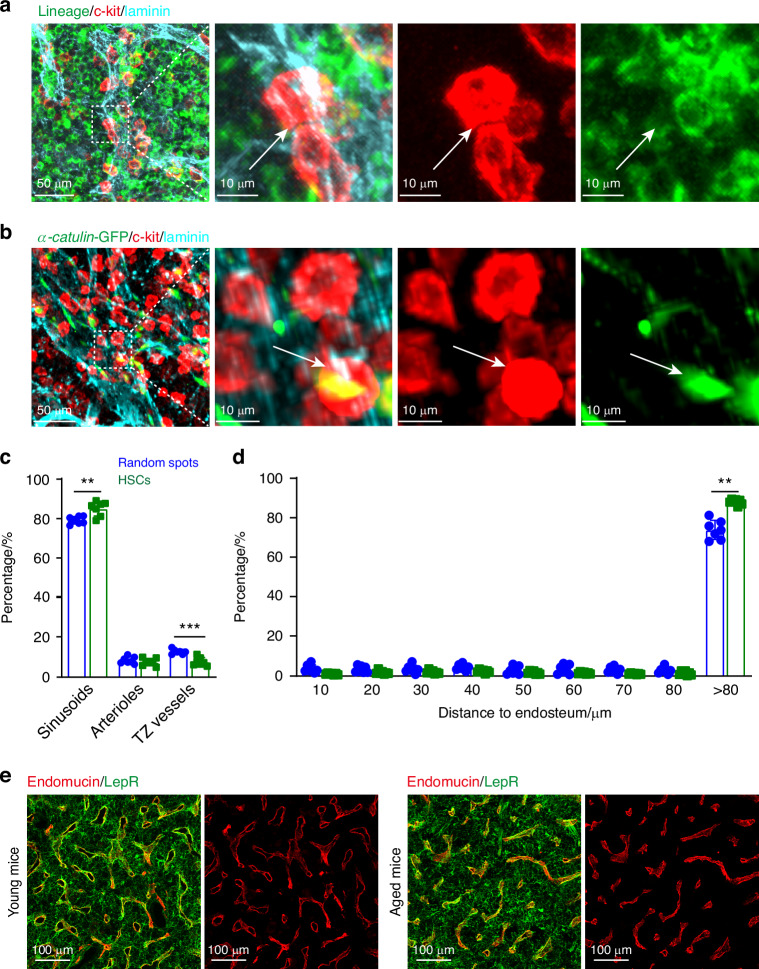
Hematopoietic stem/progenitor cells locate near the vessels in adult bone marrow. **a** 2D projected images of thick specimen. Lineage^−^c-kit^+^ hematopoietic stem/progenitor cells are in close proximity to blood vessels, identified by laminin (Cyan) staining. A single HSPC is indicated by an arrow in the higher magnification images. **b** 2D projected images of 100-µm image stacks from a cleared bone marrow plug. *α-catulin*-GFP^+^c-kit^+^ hematopoietic stem cells (HSCs) are similarly situated near vessels, identified by laminin (Cyan) staining, with a single HSC highlighted by an arrow in the magnified views. Quantitative analysis of the distance between HSCs and randomly selected spots to the nearest blood vessel (**c**) and endosteum (**d**). Data are representative of 7 experiments with one mouse per experiment. **e** 2D projected images of 50-µm image from a cleared bone hemisection. Blood vessels, identified by endomucin labeling, and LepR^+^ cells in young (2-month-old) and aged (20-month-old) wild-type mice, visualized after clearing with BABB. All images are representative of 3 experiments with one mouse per experiment per age

Hematopoietic stem/progenitor cells (HSPCs) are rare yet crucial for blood system reconstitution, primarily residing in bone marrow niches.^29,35–37^ Their precise locations and niche interactions are of significant interest to hematopoiesis researchers. For studying these cells, we utilized intact bone marrow plugs from long bones, which offer faster processing and imaging times—approximately 1–2 days shorter than bone hemisections.^38^ Our optimized method revealed that HSPCs, identified as lineage^−^c-kit^+^ cells, or LK cells, were closely associated with the vasculature (Fig. 2a). Similarly, *α-catulin*-GFP^+^c-kit^+^ hematopoietic stem cells were found in proximity to the vasculature (Fig. 2b). Quantitative analysis showed that majority of *α-catulin*-GFP^+^c-kit^+^ HSCs were closer to sinusoids than arterioles or vessels in the transition zone (significantly closer than random spots; Fig. 2c), aligning with previous findings of a peri-sinusoidal HSC niche in adult bone marrow.^29,39^ However, the use of bone marrow plugs precludes the observation of bone surface interactions, as the calcified bone is removed during the process, which hinders the visualization of endosteal interactions with bone marrow cells, including those of lymphoid progenitors.^3,40,41^ Therefore, the use of bone hemisections remains essential for such studies. Using hemisections, we demonstrated that hematopoietic stem cells (HSCs) were significantly further from the endosteum than would be expected by chance (Fig. 2d).

Bone marrow is innervated with sympathetic,^42–44^ parasympathetic,^45,46^ and sensory^47^ nerve fibers, together with glial cells.^48,49^ These peripheral nerves are known to facilitate the regeneration of various tissues, but the underlying mechanisms are not well understood.^50–52^ Nerve growth factor (NGF), highly expressed in adult bone marrow, is crucial for maintaining this innervation.^9^ To validate our optimized imaging method, we utilized *Ngf*^*mScarlet/+*^ reporter mice to pinpoint the source of marrow NGF. We found that *Ngf*-mScarlet^+^ cells are situated around both endomucin^low^ arterioles and endomucin^high^ sinusoids (Fig. S4a). These cells also express *Scf*-GFP (Fig. S4b). The peri-arteriolar cells, which include both LepR^+^*Scf*-GFP^+^ cells and smooth muscle actin (SMA) expressing cells, are predominantly *Ngf*-mScarlet^+^ (Fig. S4c). Peripheral nerves, despite their small volume fraction in bone marrow, are often overlooked in thin sections due to immunofluorescence staining limitations. Our method allowed us to clearly visualize peripherin^+^ peripheral nerve fibers along SCA-1^+^endomucin^low^ arterioles in hemisections, more efficiently and economically (Fig. S4d). This demonstrates that deep imaging is not only effective for visualizing rare cell populations like Osteolectin^+^ or NGF^+^ cells but also for capturing rare anatomic structures, such as nerve fibers, which are typically challenging to observe or quantify in standard sections.

### Deep imaging applications in other skeletal tissues and in aged mice

**Fig. 3:**
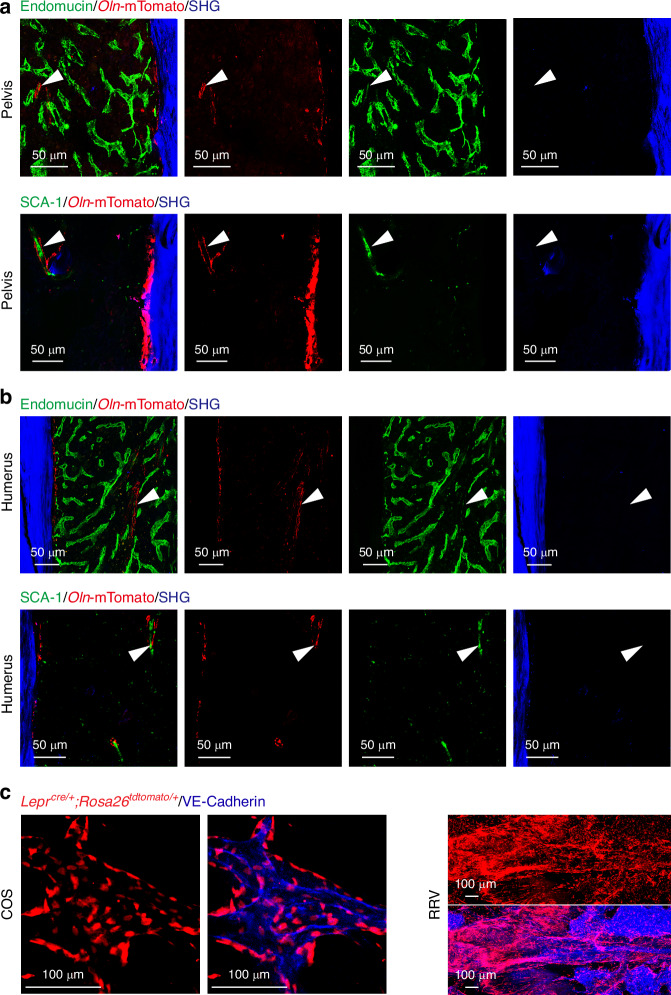
Application of the deep imaging protocol to various skeletal tissues. **a**, **b** In the pelvis and humerus bone marrow of 2-month-old *Oln*^mTomato/+^; Col1a1*2.3-eGFP mice, *Oln*-mTomato^+^ stromal cells are exclusively localized around SCA-1^+^endomucin^low^ arterioles (indicated by arrowheads), consistent with that observed in long bones. Second harmonic generation (SHG) is used to visualize the bone matrix. **c** In the skull of 2-month-old *Lepr*^cre/+^; *Rosa26*^*tdTomato*^ mice, LepR^+^ cells are observed in proximity to the endothelium, as marked by VE-Cadherin (blue), specifically in the confluence of sinuses (COS) and the rostral rhinal vein (RRV) regions. All images are representative of 3 experiments with one mouse per experiment

This optimized imaging method is applicable not only to long bones from 2- to 4-month-old mice (Fig. S1–4) and previous studies,^4,29^ but also to long bones from aged mice (≥18 months old) and various other bones within the skeletal system. In aged mice, we observed morphological changes in the femoral endothelium, which appeared more fragmented (Fig. 2e). This protocol is thus valuable for investigating aging-related alterations within the bone marrow niche, including the escalation of inflammatory responses.^53–55^ The protocol is adaptable for any bone, including calvaria and vertebrae. When applied to bones beyond long bones, we found *Oln*-mTomato expression in osteoblasts on the endosteal surface and around SCA-1^+^endomucin^low^ arterioles in the pelvis and humerus (Fig. 3a, b). LepR^+^ cells were also identified in close association with the endothelium of the rostral rhinal veins (RRV) and the confluence of sinuses (COS) in the skull (Fig. 3c). Therefore, this protocol extends beyond long bones to encompass a wide range of skeletal tissues in mice. Troubleshooting tips for the protocol are provided in Table 3.

**Table 3:** Troubleshooting of the deep imaging protocol

Step^a^	Problem	Possible reason	Solution
12	Bone samples crack or break	Decalcification may be needed for longer duration before dehydration for large bone samples such as vertebrae	Remove the surrounding muscles completely during dissection and increase the EDTA incubation time
12	Position of the bone samples when sectioning differs across samples	The bone samples were inconsistently oriented in OCT	Always embed the bone samples (step 8) in the same orientation
22	A milky suspension is seen upon transfer to BABB	Dehydration was insufficient	Extend the incubation time in methanol (step 21)
22	The organ is not sufficiently clear	Incubation in BABB was insufficient	Increase the delipidation time and number of washes with methanol or BABB incubation time
24	Air bubbles are present in the mounting system	BABB filling occurred too quickly	Use pipette tip to remove the air bubbles
25	The marrow surface is rough	Fixation was inadequate	Increase the incubation time in 4% PFA
		Decalcification was insufficient	Remove the surrounding muscles completely and increase the EDTA incubation time
25	The staining is uneven	Fixation was inadequate	Increase the incubation time in 4% PFA
		Decalcification was insufficient	Remove the surrounding muscles completely and increase the EDTA incubation time
25	There is non-specific antibody staining (especially with anti-GFP primary antibody)	Blocking was insufficient	Extend the blocking incubation time or add 0.3 M glycine to the blocking solution
25	Non-specific fluorescent crystals are present	Precipitates were present in the antibody solution	Avoid freeze-thaw cycles for the antibodies; spin the secondary antibody before use and use only the supernatant
25	Antibody staining is weak	The antibody concentration was too low	Increase the antibody concentration
		There was insufficient penetration of antibodies	Increase the incubation temperature or incubation time for all steps or consider adding up to 1% IGEPAL to the staining solution
25	Surface antibody staining is strong but inner tissue is weakly stained	The antibody concentration was too high	Decrease the antibody concentration
25	Background staining is high	The antibody concentration was too high	Decrease the antibody concentration
		The washing steps after antibody incubation were inadequate	Increase the washing temperature, total washing time, and/or the number of washes

^a^The protocol steps are described in Table 1

### Advantages and disadvantages of this method

Various methods are available for qualitative and quantitative analysis of rare cell populations in vivo. A common approach involves imaging 8–12 µm thick bone marrow sections.^39^ However, this technique’s main limitation is the difficulty in locating rare cell populations or sparse anatomical structures due to the reduced microscopy coverage, potentially leading to the omission of important areas like arterioles and nerve fibers.

**Fig. 4:**
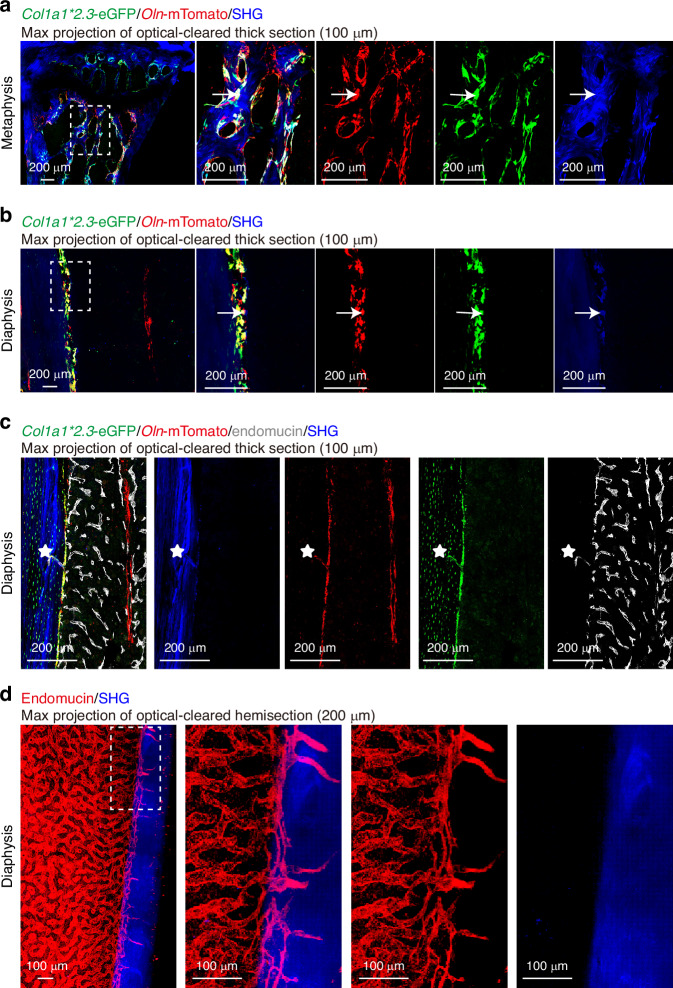
Characterization of bone marrow vasculature in long bones. In 100-µm-thick bone sections from 2-month-old *Oln*^mTomato/+^; Col1a1*2.3-*eGFP* mice, Col1a1*2.3-eGFP^+^ osteoblasts, which also express *Oln*-mTomato, were identified on both trabecular (**a**) and cortical (**b**) bone regions. **c** The transcortical blood vasculature within the cortical bone is visualized by endomucin staining (marked by white asterisk) in 100-µm-thick bone sections from the same mouse model. **d** 2D projected images of 200-µm image stack from a cleared bone hemisection. Transcortical vessels, labeled with endomucin (red), are evident in the cortical bone (blue, SHG) of the tibia from a wild-type mouse, following clearing with the BABB method. All images are representative of 3 experiments with one mouse per experiment

To overcome this, imaging thicker sections, such as 50–100 µm, can increase the imaging area and partially address these limitations.^56–60^ Optical clearing of 100-µm-thick sections revealed the presence of *Oln*-mTomato^+^ cells in both trabecular and cortical bone, marking osteoblasts on bone surfaces and osteocytes within the bone (Fig. 4a, b), similar to observations in optical-cleared hemisections (Figs. S2, 3).

**Fig. 5:**
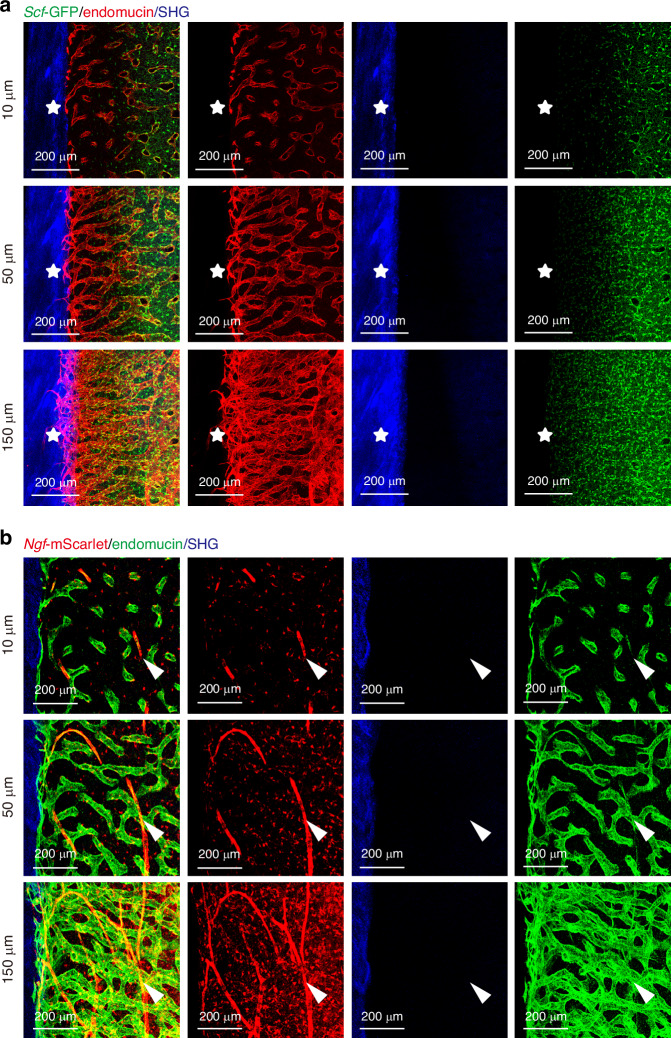
Comparative vascular imaging at various depths in murine femurs. **a** Imaging sections reveals the transcortical blood vasculature (asterisk) with thickness of 10-µm-thick sections, 50-µm-thick sections, and 2D projection of 150-µm-thick image stack from cleared bone hemisections within the femur of 2-month-old *Scf*^GFP/+^ mice. **b** Sections of femur bone marrow from 2-month-old *Ngf*^mScarlet/+^ mice, peri-arteriolar *Ngf*-mScarlet^+^ cells (arrowhead) are observed with thickness of 10 µm, 50 µm, and 2D projection of 150-µm-thick image stack from cleared bone hemisections. All images are representative of 3 experiments with one mouse per experiment

The processing time for 100-µm-thick sections is approximately 10 days, similar to optical-cleared hemisections, but with limited spatial information. Transcortical vessels in the long bones have been only recently identified due to the limitation of imaging techniques and relative scarcity comparing to highly vascularized marrow cavity.^61^ Transcortical endomucin^+^ vessels are more frequently observed in the max projection of 200-µm image stacks from optical-cleared hemisections compared to 100-µm-thick sections (Fig. 4c, d). These vessels within the cortical bone are easily identified in 150-µm-thick sections as endomucin^+^ structures, with no *Scf*-GFP^+^ perivascular stromal cells surrounding them (Fig. 5a). Additionally, arterioles, which are relatively sparse in the bone marrow cavity, are discontinuous in thin sections but more clearly visualized in 50-µm-thick sections (Fig. 5b). The max projection of 150-µm image stacks from optical-cleared hemisections offers the best structural integrity and frequency of these structures (Fig. 5b). Therefore, optical-cleared hemisections provide a unique advantage for locating rare cell populations and sparse anatomical structures with high integrity.

### Analyzing LepR^+^ cells in situ with a novel monoclonal antibody

**Fig. 6:**
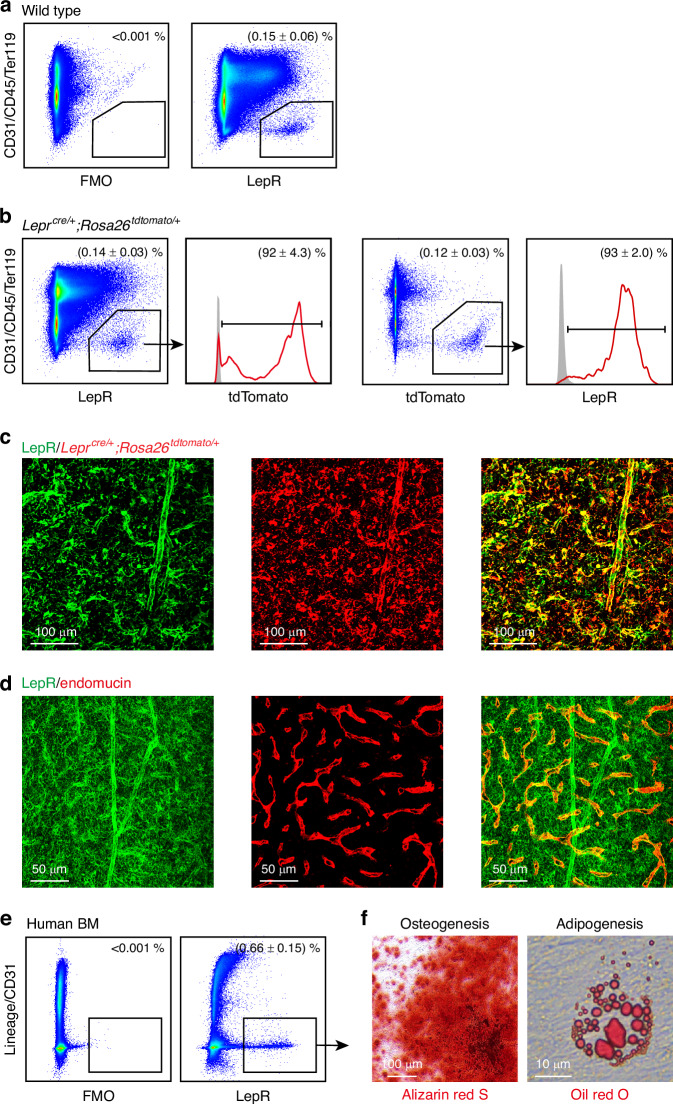
Characterization of a novel monoclonal antibody against LepR. **a**, **b** Flow cytometric analysis of enzymatically dissociated bone marrow cells from 2-month-old wild-type or *Lepr*^*cre*/+^; *Rosa26*^*tdTomato*^ mice demonstrates the identification of a putative LepR^+^ cell population using a novel anti-LepR antibody. The absence of this population in the FMO (Fluorescence Minus One) control confirms antibody specificity. **b** Anti-LepR staining overlapped with LepR^+^ cells identified by *Lepr*^*cre*/+^; *Rosa26*^*tdTomato*^ mice. Data are representative of 3 experiments with one mouse per experiment. **c** Immunofluorescence staining of a 50-µm-thick femur bone marrow section from a *Lepr*^*cre*/+^; *Rosa26*^*tdTomato*^ mouse with anti-LepR antibody. **d** Immunofluorescence staining of a 50-µm-thick bone marrow section using anti-LepR and anti-endomucin antibodies. Images are representative of 3 experiments with one mouse per experiment. **e** Application of the anti-LepR antibody in human samples was demonstrated through staining of enzymatically dissociated human bone marrow cells. Data are representative of 3 independent experiments with 3 human specimens. **f** Multilineage differentiation potential of CFU-F colonies formed by human LepR^+^ cells in panel (**e**). All Images are representative of 3 independent experiments with 3 human specimens

We have adopted this method primarily for investigating LepR^+^ cells in bone marrow, which are known to include skeletal stem cells responsible for forming most bone and fat in adult bone marrow.^11,12^ The standard antibody used for flow cytometry and immunofluorescence in thin/thick sections is a polyclonal antibody (R&D, AF497).^1,3,4,11^ However, until now, no monoclonal antibody has reliably recognized endogenous LepR, limiting research on LepR-expressing cells in situ. In partnership with Abcam, we developed and characterized a novel monoclonal antibody that efficiently stains bone marrow LepR^+^ cells. Our validation showed that (92 ± 4.3)% of bone marrow cells labeled by this anti-LepR antibody were indeed LepR^+^ (as confirmed by *LepR*^*cre*/+^; tdTomato labeling), and (93 ± 2.0)% of LepR^+^ cells were detected by this monoclonal antibody (Fig. 6a, b). This was further confirmed by immunofluorescence staining of 50-µm bone sections, where anti-LepR antibody staining coincided with tdTomato^+^ cells in *LepR*^*cre*/+^; tdTomato mice (Fig. 6c). The antibody also effectively stained perivascular LepR^+^ cells around endomucin^+^ vasculature in cleared bone sections (Fig. 6d). Importantly, this antibody cross-reacts with human LepR: flow cytometric analysis of human bone marrow using this antibody indicated that approximately (0.66 ± 0.15)% of bone marrow cells were LepR^+^ (Fig. 6e). These LepR^+^ cells from human bone marrow were capable of forming colonies and differentiating into osteogenic and adipogenic lineages (Fig. 6f), consistent with the characteristics of human skeletal stem cells.^62,63^

**Fig. 7:**
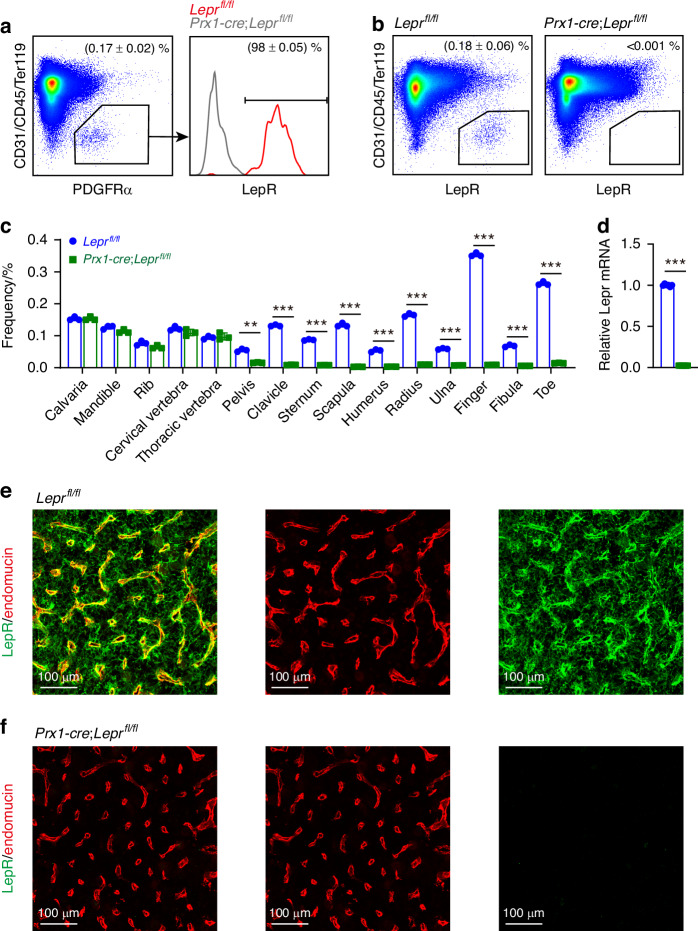
Validation of the specificity of the anti-LepR monoclonal antibody in murine bone marrow and hypothalamus. **a**–**c** Flow cytometric analysis of bone marrow cells from 2-month-old mice demonstrates the specificity of the anti-LepR antibody in various skeletal sites. Data and images are representative of 3 experiments with one mouse per experiment per genotype. **a** Most PDGFRα^+^ stromal cells in femur bone marrow were LepR^+^ from *Lepr*^*fl/fl*^ control mice but not from *Prx1*-*cre*; *Lepr*^*fl/fl*^ mice. **b** The anti-LepR monoclonal antibody labeled cells from *Lepr*^*fl/fl*^ control mice but not from *Prx1*-*cre*; *Lepr*^*fl/fl*^ mice. **c** The frequency of cells stained with the anti-LepR antibody in bone marrow from various skeletal sites was assessed, including the calvaria, mandible, rib, cervical and thoracic vertebrae, pelvis, clavicle, sternum, scapula, humerus, radius, ulna, fingers, fibula, and toe. **d** *Lepr* mRNA levels were determined by qRT-PCR in PDGFRα^+^ CD31^−^CD45^−^Ter119^−^ femur bone marrow cells from *Prx1*-*cre*; *Lepr*^*fl/fl*^ and *Lepr*^*fl/fl*^ littermate control mice. Data are representative of 6 experiments with one mouse per experiment per genotype. **e–h** Representative staining of anti-LepR antibody in *Lepr*^*fl/fl*^ control mice (**g**) and *Prx1*-*cre*; *Lepr*^*fl/fl*^ mice (**h**) hypothalamus. Images are representative of 3 experiments with one mouse per experiment per genotype

To verify the specificity of our anti-LepR monoclonal antibody, we used the *Prx1*-*cre* allele to conditionally delete *Lepr* in limb bone marrow stromal cells, excluding the axial skeleton. Flow cytometric analysis of femoral bone marrow from *Prx1*-*cre*; *Lepr*^*fl/fl*^ mice showed a significant reduction in LepR expression among PDGFRα^+^ stromal cells compared to controls (Fig. 7a, b). We then examined additional bone marrow compartments across various skeletal regions and observed significant LepR deletion in stromal cells from the pelvis, clavicle, sternum, scapula, humerus, radius, ulna, finger, fibula, and toe bone marrow (Fig. 7c). No deletion was detected in the calvaria, mandible, rib, or cervical and thoracic vertebrae. Quantitative RT-PCR (qRT-PCR) confirmed the reduction of *Lepr* transcripts in sorted stromal cells from the femoral bone marrow of *Prx1*-*cre*; *Lepr*^*fl/fl*^ mice (Fig. 7d). Immunofluorescence staining further demonstrated a predominant depletion of LepR expression in the femoral bone marrow (Fig. 7e, f), confirming the specificity of the monoclonal antibody.

**Fig. 8:**
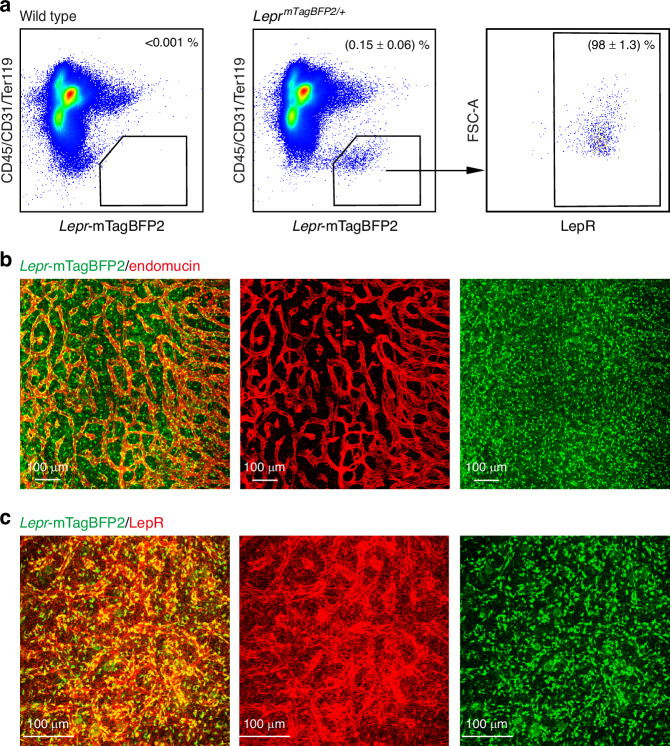
Characterization of a *Lepr*^*mTagBFP2*^ reporter allele. **a** Flow cytometric analysis of enzymatically dissociated femur bone marrow cells from 2-month-old wild-type and *Lepr*^*mTagBFP2*/+^ mice, demonstrating that most *Lepr*-mTagBFP2^+^ stromal cells were LepR^+^. 3 mice from 3 independent experiments. Deep imaging of femur bone hemisections from 2-month-old *Lepr*^*mTagBFP2*/+^ mice shows that *Lepr-*mTagBFP2^+^ cells were perivascular, locating on endomucin^+^ vessels (**b**), and *Lepr-*mTagBFP2^+^ cells were all LepR^+^ (**c**). **d** *Lepr*-mTagBFP2^+^ cells are observed in the hypothalamus of *Lepr*^*mTagBFP2*/+^ mice. Data and images are representative of 3 experiments with one mouse per experiment

We generated a *Lepr-mTagBFP2* (*Lepr*^*mTagBFP2*^) knock-in reporter allele to facilitate the investigation of LepR^+^ cells, allowing for the specific visualization of these cells (Fig. S5a–c). By qRT-PCR, *Lepr* transcripts were enriched in flow cytometrically isolated bone marrow *Lepr*-mTagBFP2^+^ cells, but nearly undetectable in whole bone marrow cells or *Lepr*-mTagBFP2^−^ cells (Fig. S5d). Immunofluorescence staining of the brain section revealed *Lepr*-mTagBFP2^+^ neurons in the hypothalamus (Fig. S5e), consistent with previous report using the *Lepr*^*Cre/+*^ allele.^64^ Flow cytometric analysis of enzymatically dissociated bone marrow cells showed that (0.15 ± 0.06)% of bone marrow cells were *Lepr*-mTagBFP2^+^, with (98 ± 1.3)% co-expressing LepR (Fig. 8a). Deep imaging of bone hemisections confirmed that *Lepr*-mTagBFP2^+^ cells are predominantly perivascular, closely associated with endomucin^+^ vessels (Fig. 8b). This method ensures antibody penetration to a depth of at least 500 µm without significant reduction in signal intensity (Fig. S6a, b) or loss of antibody labeling specificity (Fig. S6c). The staining pattern of *Lepr*-mTagBFP2 in bone marrow sections overlapped with that of LepR antibody staining, corroborating the flow cytometric data (Fig. 8c). Furthermore, it is evident that bone marrow stromal cells, as indicated by *Lepr*-mTagBFP2 or *Scf*-GFP labeling, are not present on endosteal surfaces (Fig. S6b, c).

### Adaptations of the protocol for other experimental systems

**Fig. 9:**
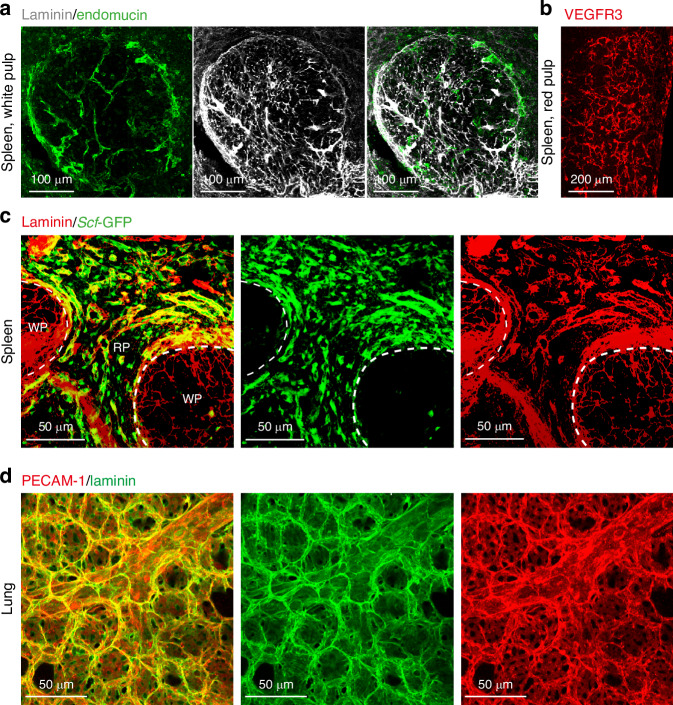
Deep imaging of the spleen and lung vasculature post optical clearing. **a** Deep imaging of the white pulp (WP) vasculature in a wild-type mouse spleen, visualized with antibodies against laminin (white) and endomucin (green). **b** Deep imaging of VEGFR3^+^ sinusoidal vessels in the red pulp (RP) of a wild-type mouse spleen. **c** Deep imaging of vasculature in the white pulp and red pulp of a spleen from a *Scf*^GFP/+^ mouse. **d** Deep imaging of endothelial cells and the basement membrane in the alveolus pulmonis of a wild-type mouse lung, using antibodies against laminin (green) and PECAM1 (red). Images are representative of three experiments with one mouse per experiment

Deep imaging has been extensively utilized by research groups to visualize the localization of diverse cell populations in various tissues. It has been particularly instrumental in examining how environmental and genetic factors influence cell distribution and proliferation in vivo.^65–70^ This protocol is not limited to bone marrow; it can be adapted to study other hematopoietic tissues, including the spleen.^71^ For instance, in the spleen, Vegfr3^+^ endothelial cells are predominantly found in the red pulp (Fig. 9a, b), and in *Scf*^*GFP/+*^ mice, *Scf*-GFP^+^ stromal cells are also mainly localized to the red pulp (Fig. 9c). Furthermore, this protocol allows for the visualization of PECAM1^+^ endothelial cells with detailed structural features in cleared lung tissue (Fig. 9d), consistent with previous findings.^71,72^ These applications demonstrate that the protocol is effective for soft tissues beyond the skeletal system and is likely applicable to a wide range of organs and tissues.

## Discussion

Optical clearing techniques, first introduced over a century ago by Spalteholz,^73^ have revolutionized volumetric imaging by overcoming the limitations imposed by light scatter in thick tissues. This method is crucial for organ-level imaging, where the working distance of the microscope and scattered light would otherwise hinder detailed visualization. In recent years, advanced optical imaging methods have been employed across various fields to study the three-dimensional architecture of cells within different organs.^29,57,66,74–77^ These studies have utilized fluorescence-based reporters through antibody labeling or transgenic mice, combined with various optical clearing approaches and imaging techniques such as confocal or two-photon microscopy. The successful implementation of a high-resolution deep imaging method requires the development of a customized antibody labeling and optical clearing pipeline tailored to the tissue of interest that is time- and cost-effective as well as providing acceptable results.

In the past decade, advancements in optical clearing techniques have enabled the immunofluorescence staining of opaque tissues, with iDISCO,^38,66,70,74,78^ PEGASOS,^77,79,80^ and other examples.^57,81^ A comparison of these methods is detailed in Table 4. The assay we present offers the benefits of a shorter processing time, approximately 11 to 12 days, and is more cost-effective for high-resolution immunofluorescence staining and imaging. However, it is not without limitations. Unlike traditional bone section staining, our method permits only one staining combination per bone sample. Additionally, certain chemical stains, including Oil Red O,^82^ are incompatible with our protocol due to the delipidization phase. Compatibility of antibody and fluorophore combinations must be verified, as with iDISCO and other clearing techniques, to ensure they withstand the fixation, permeabilization, and clearing processes. Despite these considerations, the costs associated with deep imaging through optical clearing remain higher than those of traditional section staining methods.

**Table 4:** A comparison of several methods for generating optically clear tissues for immunofluorescence staining

Method	Applicable tissues	Compatible with immunostaining?	Preservation of endogenous fluorescent proteins?	Tissue clearing period	Advantages	Limitations
**Thick sections (100–150** **µm)**^56–60^	Bone marrow, spleen, muscle, heart, gut, skin, lung, etc.	Yes	Yes	Not applicable	Simple to implement; inexpensive; reduces background signals; non-toxic	Spatial information is limited; difficult to study rare populations
**BABB**^4,29,67,71,76,88^	Bone marrow, spleen, muscle, heart, gut, skin, lung, etc.	Yes	Yes	Not applicable	Simple to implement; inexpensive; reduces background signals; non-toxic	Spatial information is limited; difficult to study rare populations
**PEGASOS**^77^	Bones, brain, muscles, heart, lung, kidney, liver, pancreas, spleen, muscle, stomach, intestine, skin	Yes	Yes	~12–14 days for hard tissues, ~7 days for soft tissues	Strong clearing capability for both soft and hard tissues; no need to immunostain endogenous fluorescent proteins	Soft tissues undergo variable shrinkage; complicated protocol
**Eci**^65,89–91^	Kidney, heart, calvarial and long bones	Yes	No	~3 days	Non-toxic clearing reagent	Difficult long-term storage
**iDISCO**^38,66,70,74,78^	Brain, heart, vertebral arteries, lung, liver, prostate	Yes	No fluorescence is quenched	>3 h for small organs, 1–2 weeks for the whole mouse embryo and complex adult organs	Strong clearing capability for soft tissues	Toxic and thus careful handling is required; tissues are fragile; not all epitopes are applicable; soft tissues undergo shrinkage
**Bone CLARITY**^75^	Bone	Yes	Yes	28 days	Imaging depth of up to 1.5 mm for the epiphysis of long bones; integrity of the bone marrow is retained	Poor penetration of relatively large antibodies

Our optimized imaging methods are designed for efficiency and can be applied across a spectrum of organs, regardless of age. These techniques enable us to elucidate the intricate architecture and cellular components of tissues, and crucially, to analyze the spatial relationships among various cellular elements. This includes the ability to examine transcortical vessels and other cellular interactions both quantitatively and qualitatively. The ongoing evolution of optical clearing techniques and volumetric imaging, particularly when combined with diverse antibody labeling, is set to significantly improve our understanding of rare cell populations and their spatial dynamics within organs. This advancement is expected to provide deeper insights into tissue behavior under homeostatic, regenerative, and pathological states.

## Materials and methods

### Mice

All mouse experiments complied with all relevant ethical regulations and were performed according to protocols approved by the Institutional Animal Care and Use Committee at the National Institute of Biological Sciences, Beijing. All mice were maintained on a C57BL/6 J background, including *Lepr*^*cre*^ (JAX: 008320^64^)*, Prx1*-cre (JAX: 005584), *Lepr*^*flox*^ (JAX: 008327), *Rosa26*^*tdTomato*^ (Ai14) (JAX: 007914^83^), *Rosa26*^*TriGFP*^ (Ai47^84^), Col1a1*2.3-*eGFP* (JAX: 013134^85^), *Oln*^mTomato^ (ref. ^4^), *Ngf*^mScarlet^ (JAX: 039432^9^), and *Scf*^*GFP*^ (JAX: 017860^1^).

To generate *Lepr*^*mTagBFP2*^ mice, Cas9 mRNA, single guide RNA, and targeting vector containing 3 × *mTagBFP2* inserted before stop codon of the *Lepr* gene were co-microinjected into C57BL/6 J zygotes. The coding sequence for the monomeric blue fluorescent protein (mTagBFP2) was as described.^86^ Chimeric mice were genotyped by restriction fragment length polymorphism analysis and insertion of the 3 × *mTagBFP2* sequence into the correct locus was confirmed by Southern blotting and sequencing of the targeted allele. Founders were mated with C57BL/6 J mice to obtain germline transmission then backcrossed with wild-type C57BL/6 J mice for at least three generations before analysis. Genotyping primers for genotyping *Lepr*^*mTagBFP2*^ mice were Lepr-Com-G2, 5ʹ-CTT TCT CTA GCA GCT CCT GGG AG-3ʹ; Lepr-mTagBFP2-G3, 5ʹ-CAG TAG ACT AAA ATT CGT CGC TC-3ʹ; and Lepr-WT-G1, 5ʹ-GAG ACC TTC CCC AAG TAT CTT GG-3ʹ.

### Bone hemisection and thick section preparations

Mice were euthanized by cervical dislocation. Intact mouse femurs and tibias were carefully dissected by removing all surrounding tissues without causing any damage to the bones. The bones were then transferred to 15-mL centrifuge tubes filled with ice-cold (i.e., placed on ice for ~15 min) 4% PFA (Solarbio, P1110) and were incubated at 4 °C on a rotator for 6 h. After fixation, the bone samples were washed twice with PBS (at least 10 mL PBS per wash) under constant agitation in a 15-mL centrifuge tube at 4 °C. The bone samples were then incubated with 15 mL ice-cold EDTA (0.5 mol/L, dissolved in water at pH 8.0; Solarbio, E8040) for decalcification (this step can be optional). The bones were then washed twice with PBS and incubated with 10 mL ice-cold 30% sucrose (Macklin, S818046) solution under constant agitation on a rotator at 4 °C for 12 h, followed by OCT (Sakura, 4583) embedding.

The embedded long bones were sectioned longitudinally at a thickness of 50–100 µm using a new precooled, low-profile microtome blade until the sinus and the full longitude of the long bone were fully exposed, to generate the bone hemisections (over 500 µm in thickness from adult mice at 2–4 months of age). For experiments using bone sections, OCT-embedded femurs were sectioned using the CryoJane tape-transfer system (Leica Biosystems). Sections were blocked in PBS with 5% donkey serum (Jackson Immuno, 017-000-121) for 1 h and then stained with primary antibodies overnight.

### Flow cytometry and in vitro differentiation assay

For flow cytometric analysis, bone marrow cells from mice were flushed using Ca^2+^- and Mg^2+^-free Hanks’ Balanced Salt Solution (HBSS) (Beyotime, C0218) with 2% bovine serum (Gemini, 900-108) and then enzymatically dissociated as described.^31^ Anti-LepR (Abcam, ab318272), anti-CD45 (30F-11, Invitrogen, 11-0451-85), anti-CD31 (390, Invitrogen, 11-0311-85), and anti-TER119 (TER119, BioLegend, 116206) were used to isolate LepR^+^ stromal cells that were negative for hematopoietic and endothelial markers. Anti-PDGFRα-biotin (eBioscience, clone APA5, 1:200), anti-CD45 (30F-11, Invitrogen, 11-0451-85), anti-CD31 (390, Invitrogen, 11-0311-85), and anti-TER119 (TER119, BioLegend, 116206) were used to isolate PDGFRα^+^ stromal cells that were negative for hematopoietic and endothelial markers. For the analysis of human bone marrow, samples were isolated from patients undergoing hip arthroplasty, and LepR^+^ stromal cells were isolated using anti-LepR (Abcam, ab318272), CD31 (WM59, BioLegend, 303104), CD45 (2D1, BioLengend, 368508), CD45R (RA3-6B2, BioLegend, 103206), CD41 (HIP8, BioLegend, 303704), CD61 (VI-PL2, BioLegend, 336404), CD71 (MEM-75, Abcam, AB239251), and CD235ab (HIR2, BioLegend, 306610).

All human studies were conducted in accordance with the official ethical guidelines and protocols approved by the Ethics Committee of the Peking University People’s Hospital (2024-z063). For analysis of human bone marrow cells, fresh bone marrow was drawn from the proximal femur of patients who underwent total hip replacement in Peking University People’s Hospital. We collected discarded bone marrow during femoral medullary reaming in total hip replacement. CFU-F assays were performed similarly as described previously for mouse bone marrow stromal cells,^31^ followed by osteogenic or adipogenic differentiation with StemPro Differentiation Kits (Invitrogen, A1007201 for osteogenic differentiation; Invitrogen, A1007001 for adipogenic differentiation). Osteogenic and adipogenic differentiation were assessed using Alizarin Red S (OriCell, OILR-10001), and Oil Red O staining (Sigma-Aldrich, O0625).

### Immunofluorescence staining

The bone hemisections were transferred to 1.5-mL microcentrifuge tubes, placed on ice, and washed twice with 1.5 mL of PBS to remove the OCT completely. The PBS solution was removed, and the hemisections were incubated with 5% donkey serum in 0.5% PBST for 6 h at room temperature with shaking to reduce non-specific background signal. After that, the hemisections were transferred to a new 0.6-mL centrifuge tube using forceps, and 550 μL of primary antibody staining solution (blocking solution with primary antibody cocktail) was added. The hemisections were then incubated with shaking at room temperature for 3 days. After the primary antibody incubation, the hemisections were transferred to a new 1.5-mL centrifuge tube with forceps and were washed three times with PBS for 15 min each at room temperature. A fourth wash with PBS was carried out overnight with shaking at room temperature. The washed hemisections were then transferred to a new 0.6-mL centrifuge tube using forceps, and 550 μL of secondary antibody solution (blocking solution with secondary antibody cocktail) was added. The hemisections were then incubated with shaking at room temperature in the dark for 3 days, followed by another three washes as described above in a 1.5-mL microcentrifuge tube. For bone sections, primary antibodies were stained overnight, followed by three washes for 20 min each, and secondary antibodies were incubated for 1 h, followed by three washes for 20 min each.

### Optical clearing of stained tissues

At least 24 h before the optical clearing steps, the BABB buffer needs to be prepared. A 50-mL BABB mixture [benzyl alcohol (Macklin, B802546) /benzyl benzoate (Aladdin, B400547) = 1:2 (vol/vol)] is prepared in a glass cylinder and then is added to a 50-mL centrifuge tube containing 5 g of aluminum oxide (Macklin, A800193). The centrifuge tube is sealed with Parafilm and wrapped with aluminum foil to block out any light. The mixture is incubated with shaking for (24–48)h. Before the BABB is used for optical clearing, the mixture is centrifuged at 800 × *g* for 15 min at room temperature.

The hemisections are then dehydrated by methanol incubation for 5 min and repeated three times, with 1.5 mL of methanol for each wash. After dehydration, the hemisections are cleared with one or two washes with BABB. The hemisections are incubated in BABB with shaking at room temperature in the dark for several hours to overnight. The BABB is then replaced with fresh BABB, and the filled microcentrifuge tube is wrapped in aluminum foil. The hemisections can be stored at 4 °C in BABB in the dark for up to at least 3 months before imaging.

### Preparing mounting slide for image acquisition

After optical clearing, the hemisection sample in BABB is mounted within the custom quartz mold. The design of this mounting system is shown in Fig. S1. Silicone gel (Soudal, Silirub 2/S) was drawn up into a 10-mL syringe and carefully squeezed out into the mold. The hemisection sample was placed on top of the fresh silicone. Importantly, the sample was oriented with the marrow cavity facing up and was adjusted to be as close to horizontal (i.e., parallel to the bottom of the dish) as possible. A few drops of BABB were added to the silicone pad, and the silicone foundation was allowed to solidify at room temperature for 30 min before the pool was filled with fresh BABB to immerse the hemisection sample. The mounted section was then used for image acquisition. Note that the stained and cleared bone hemisections can be preserved for up to at least 3 months without any notable loss of fluorescence, if they are stored in BABB at 4 °C in the dark.

### Data acquisition and analysis

Bone hemisection samples are thick (>500 µm), which can lead to substantial background fluorescence. To image the skeletal architecture in three dimensions at high resolution with minimal background signal, we used a Leica Stellaris or Leica SP8 resonant confocal laser-scanning microscope with z-stack scanning and tiling to obtain sequential depth images. Laser lines used were at 488, 561, and 633 nm. The Leica confocal microscope allowed us to image multiple channels efficiently and minimized the acquisition time. We used an 8-kHz resonant tandem scanner with both HyD (hybrid detector) and PMT (photomultiplier tube) detectors and used 3× line averaging to optimize the signal-to-noise ratio. Deep imaging of the stained tissue requires a long working distance objective, with a refractive index (1.56) matching the optimum for the clearing agent BABB. We chose the HCX APO L20×/0.95 BABB immersion objective with a 1.95-mm working distance to achieve deep imaging of thick tissues in the presence of BABB. Pixel size was set as 0.867 × 0.867 µm, pinhole size was 46.9 µm, and each z-step was 2 µm. The Alexa 488 channel can sometimes result in a high background fluorescence after tissue clearing. Optimal acquisition settings and antibody combinations were chosen to balance the imaging time and imaging quality (adequate lateral and axial resolution, high signal-to-noise ratio for further quantification and analysis). For tissues such as muscle that have a high background signal, multispectral imaging can be used to improve the signal-to-noise ratio.^87^

After image acquisition, the tiled z-stack images were converted into “.ims” or “.aivia.tif” format using Imaris x64 10.1.0 (Bitplane) or Aivia 12.1.0 respectively, followed by three-dimensional rendering, snapshot generation, and quantification. Aivia 12.1.0 or Bitplane Imaris x64 10.1.0 was installed on a ThinkStation P920 Workstation with Dual Intel Xeon Platinum 8160 T CPU @ 2.10 GHz, 1.00 TB RAM, and an NVIDIA Quadro RTX 8000 graphics card. We first set the blood vessels or bone as different surfaces based on the antibody staining or SHG signal using the surface function of Imaris. Based on Laminin antibody staining and morphological differences, arteries and sinusoids were manually labeled and further applied to the machine-learning process with the ‘Machine Learning Segmentation’ with ‘All Channels’ function in Imaris to identify arteries and sinusoids in the samples. As previously described,^29^ the outer 20% of the bone marrow by volume was set as transition zone (TZ). The bone marrow cavity and TZ vessel surface were labeled every 5-10 slices. Next, we saved the current surface with Export Scene function and load the original ‘.ims’ file for further analysis. The ortho slicer function in Imaris was then used to identify individual *α-catulin*-GFP^+^c-kit^+^ hematopoietic stem cells (HSCs) across the slices in the large three-dimensional images. The HSCs were determined based on a round morphology and a co-expression of GFP and c-kit. The spot function in Imaris was then applied to set these identified HSCs as spots, with “object-object statistics” in the algorithm setting options selected with default parameters. We created a randomly located spot assembly by using a Matlab script, in which we could set the same number of spots of 6μm diameter and spread them in the exact same volume of the crop which we analyzed the number of HSCs. We then created a Spot subject in the Imaris file which represented the randomly spread spots using Matlab. After all these, we imported all the surfaces and spots into the original Imaris file and entered the ‘Vantage’ interface. Finally, we exported the distance of every single spot to the different surfaces in ‘details’, compared the differences, and calculated the percentages with the object-object statistics.

## Supplementary information

Supplementary Figure 1

Supplementary Figure 2

Supplementary Figure 3

Supplementary Figure 4

Supplementary Figure 5

Supplementary Figure 6

Supplementary figures captions
