# Supplementary figures and images for: Deep imaging of LepR+ stromal cells in optically cleared murine bone hemisections
Source: Bone Res. 2025 Jan 13;13:6. doi: 10.1038/s41413-024-00387-9 (PMC11725602; doi:10.1038/s41413-024-00387-9)

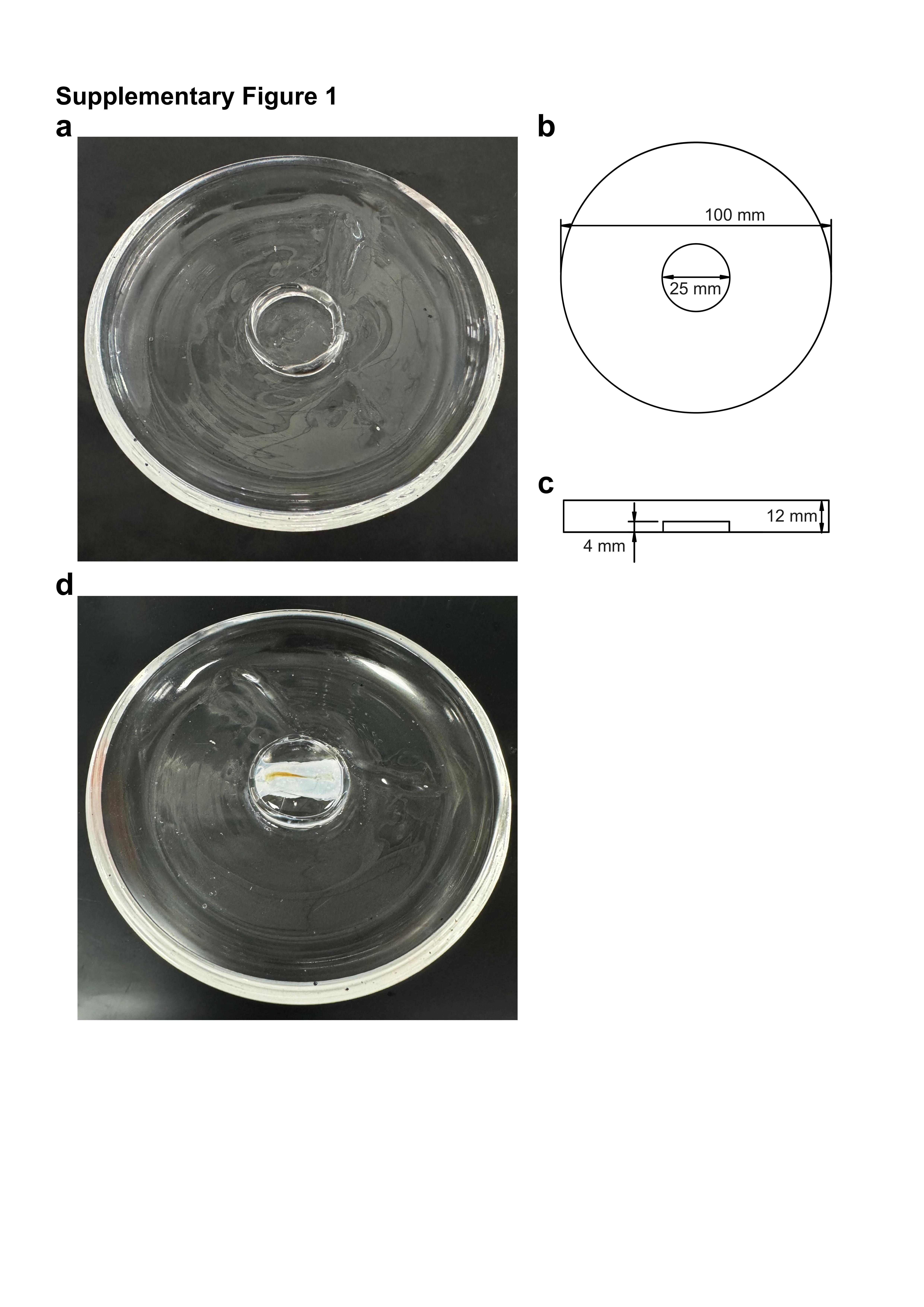

Supplement: Supplementary file 1 — Supplementary Figure 1 [file 41413_2024_387_MOESM1_ESM.jpg]

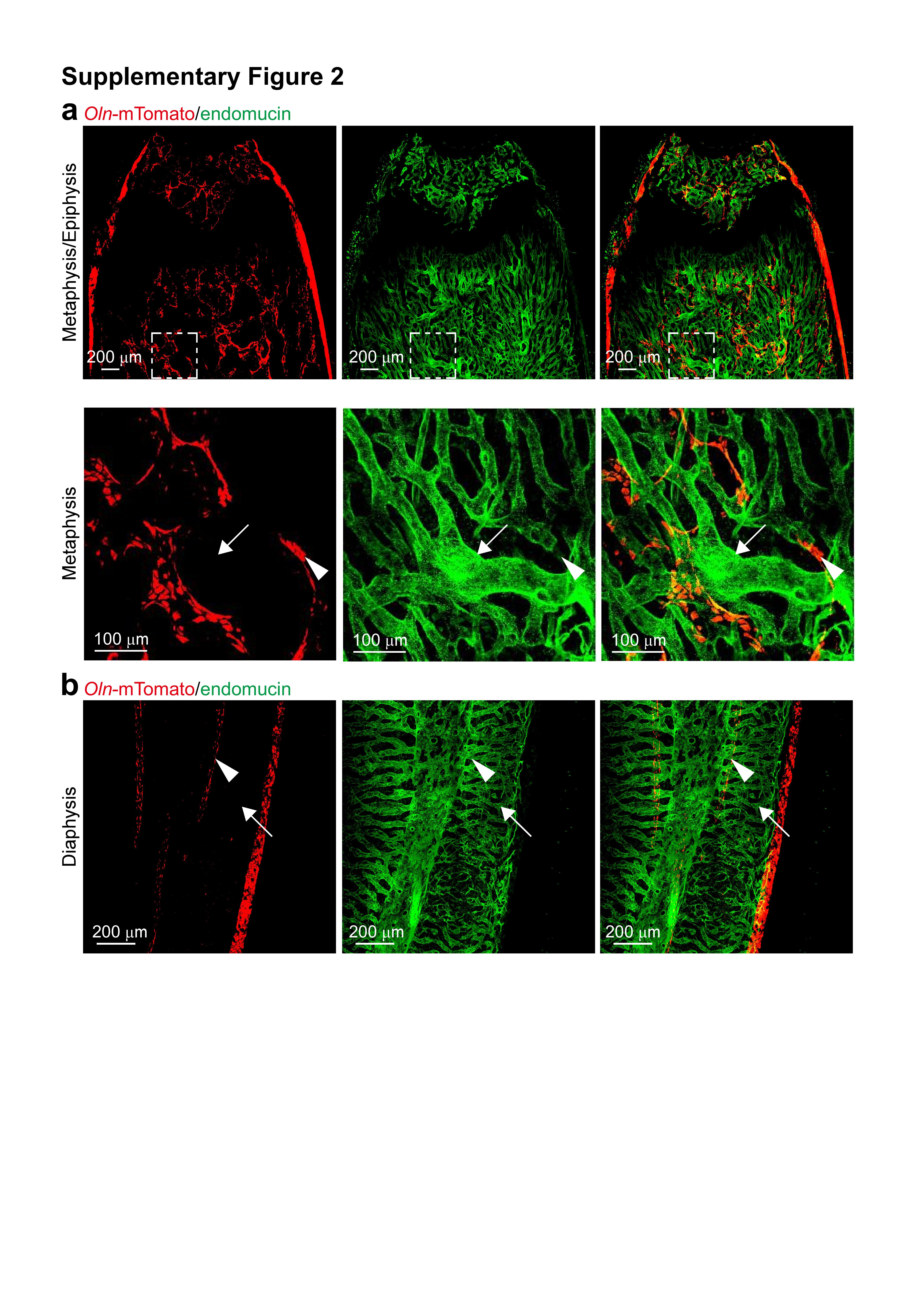

Supplement: Supplementary file 2 — Supplementary Figure 2 [file 41413_2024_387_MOESM2_ESM.jpg]

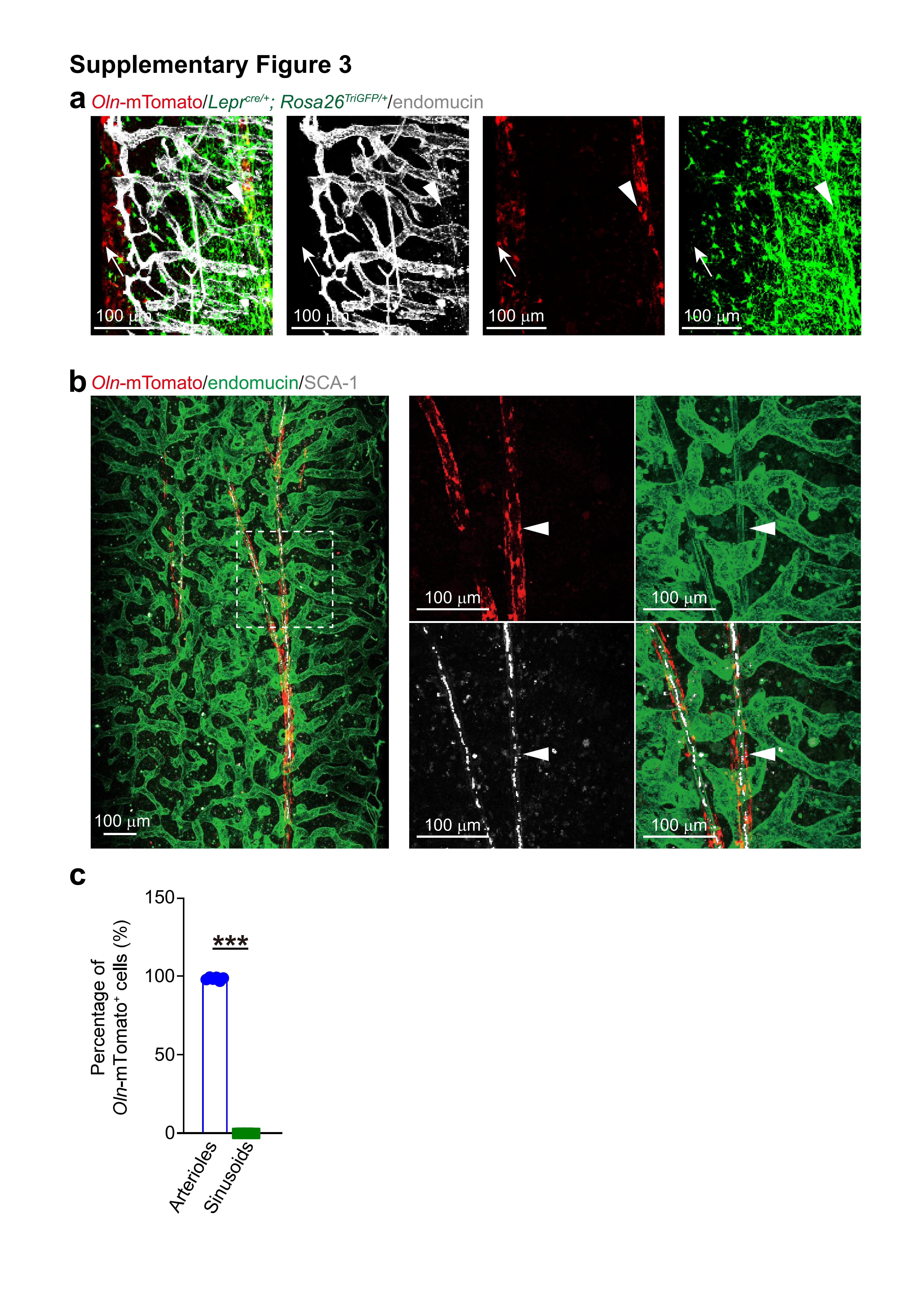

Supplement: Supplementary file 3 — Supplementary Figure 3 [file 41413_2024_387_MOESM3_ESM.jpg]

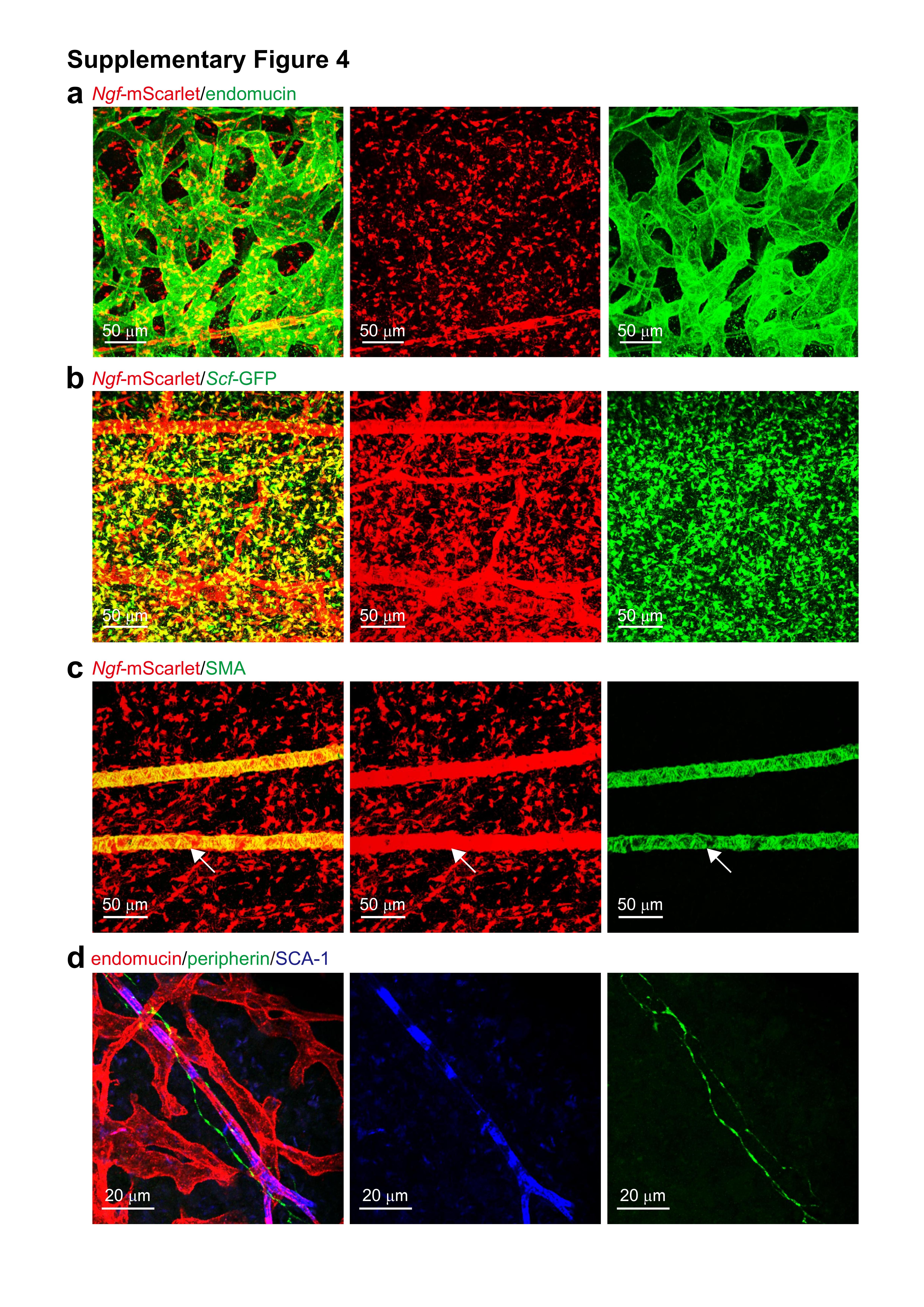

Supplement: Supplementary file 4 — Supplementary Figure 4 [file 41413_2024_387_MOESM4_ESM.jpg]

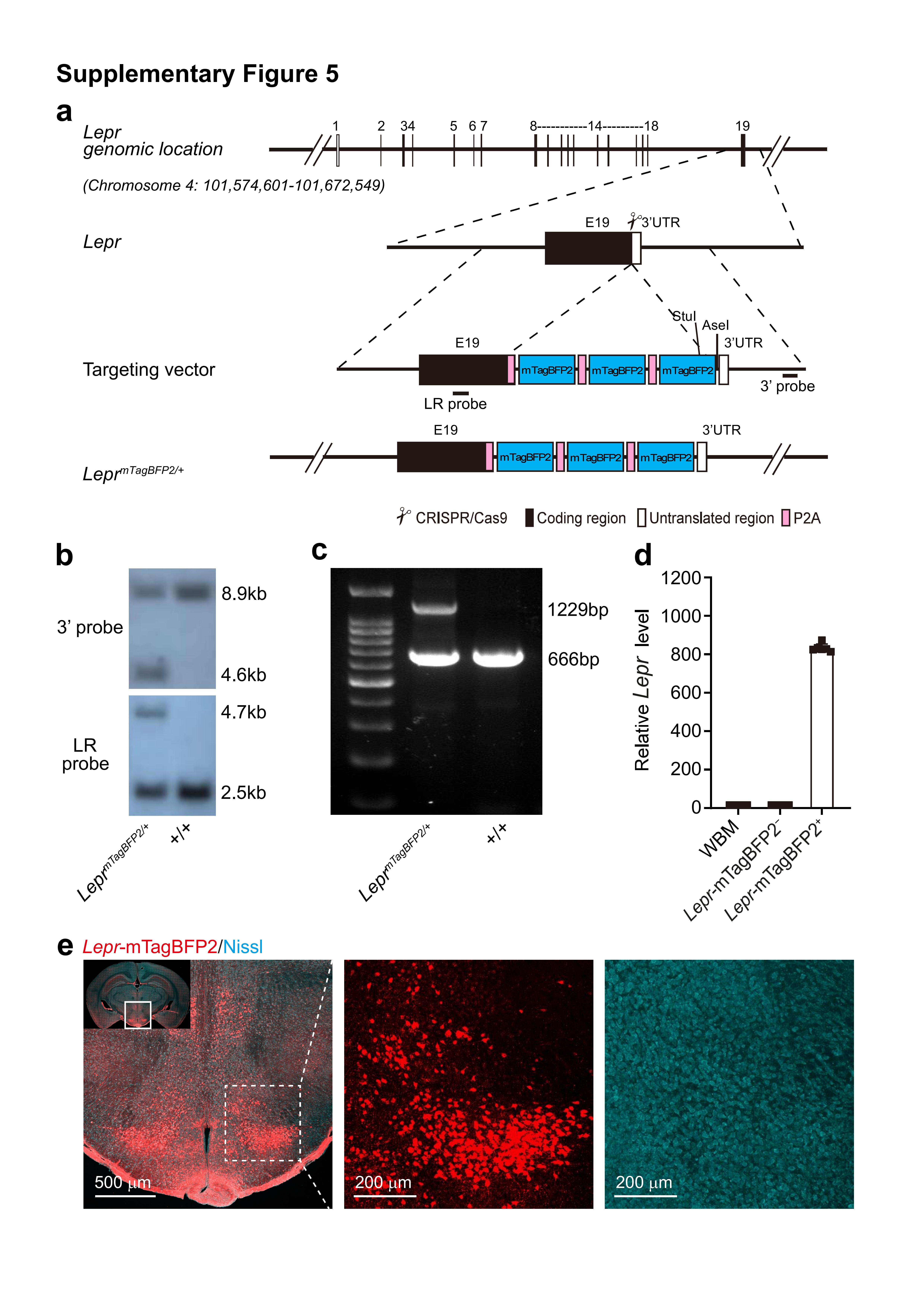

Supplement: Supplementary file 5 — Supplementary Figure 5 [file 41413_2024_387_MOESM5_ESM.jpg]

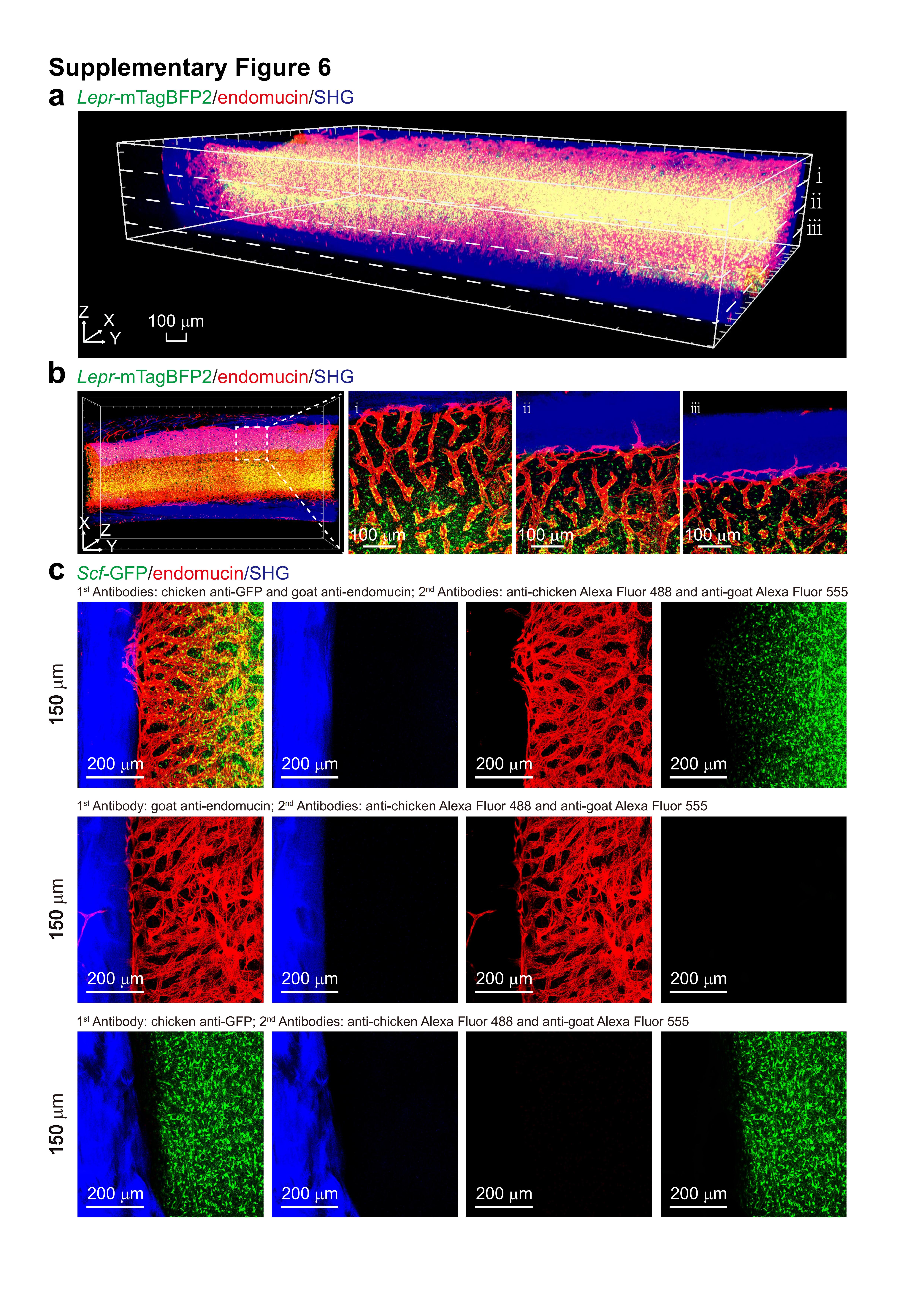

Supplement: Supplementary file 6 — Supplementary Figure 6 [file 41413_2024_387_MOESM6_ESM.jpg]
