# Supplementary material for: Deep imaging of LepR+ stromal cells in optically cleared murine bone hemisections
Source: Bone Res. 2025 Jan 13;13:6. doi: 10.1038/s41413-024-00387-9 (PMC11725602; doi:10.1038/s41413-024-00387-9)
Supplement: Supplementary file 7 — Supplementary figures captions [file 41413_2024_387_MOESM7_ESM.docx]

**Supplementary Figure 1. Supplementary Figure 1. Bone hemisection mounting technique on a quartz mold.** (**a**) Custom quartz mold for specimen placement, exemplified with bone hemisections. (**b**) Top-down view of the mold, delineating the specimen arrangement and alignment. (**c**) Lateral view of the mold, highlighting the geometric form and precise dimensions. (**d**) Representative image of a bone hemisection secured within the mold using silicone.

**Supplementary Figure 2. *Oln*-mTomato^+^ cells include osteoblasts and stromal cells.** (**a**, **b**) In 2-month-old *Oln*^mTomato/+^ mice, *Oln*-mTomato^+^ cells are absent from the endomucin-rich sinusoids in the metaphysis (arrow in **a**) and diaphysis (arrow in **b**). These cells are identified as osteoblasts in the metaphysis (arrowhead in **a**) and peri-arteriolar stromal cells in the diaphysis (arrowhead in **b**). Images are representative of 3 experiments with one mouse per experiment.

**Supplementary Figure 3. *Oln*-mTomato^+^ stromal cells are exclusively peri-arteriolar in adult bone marrow.** (**a, b**) In femur bone marrow from 2-month-old *Lepr*^cre/+^; *Rosa26^TriGFP/+^*; *Oln^mTomato/+^* mice, *Oln*-mTomato^+^LepR^+^ stromal cells were exclusively peri-arteriolar (arrowhead in **a, b**). Endosteal osteoblasts (arrow in **a**), which are LepR^−^, are also *Oln*-mTomato^+^. (**c**) All *Oln*-mTomato^+^ cells were peri-arteriolar. 6 mice from 6 independent experiments. All images are representative of 3 experiments with one mouse per experiment.

**Supplementary Figure 4. *Ngf*-expressing cells are mostly *Scf*-expressing LepR^+^ cells in adult bone marrow**. (**a**) In femur bone marrow from 2-month-old *Ngf*^mScarlet/+^ mice, *Ngf*-mScarlet is expressed by stromal cells surrounding endomucin^low^ arterioles as well as endomucin^high^ sinusoids. (**b**) Most *Ngf*-mScarlet^+^ stromal cells are *Scf*-GFP^+^. (**c**) *Ngf*-mScarlet is expressed by a much smaller number of SMA^+^ smooth muscle cells relative to LepR^+^ cells in femur bone marrow (arrow in **c**). (**d**) Peripheral nerve fibers (labeled with peripherin) are localized along the SCA-1^+^endomucin^low^ arterioles in bone marrow from 2-month-old wild-type mice. All images are representative of 3 experiments with one mouse per experiment.

**Supplementary Fig 5. Generation of the *Lepr^mTagBFP2^* mouse allele. (a)** Schematic representation of the mouse *Lepr* gene modification, integrating a 3x mTagBFP2 cassette between exon 19 and the 3′ untranslated region, without disrupting conserved intronic sequences. Open boxes denote untranslated regions, while black boxes represent translated regions of *Lep*r. (**b**) Identification of the F1 generation mice by Southern blotting using the 3′ and LR probes indicated in panel **a**. (**c**) PCR genotyping of genomic DNA confirmed germline transmission of the *Lepr^mtagBFP2^* allele. Mice were backcrossed onto a C57BL/6J background for at least three generations prior to analysis. (**d**) Quantitative RT-PCR (qRT-PCR) analysis of *Lepr* expression in whole bone marrow (WBM) cells, *Lepr-*mTagBFP2^−^CD45^−^Ter119^−^CD31^−^ stromal cells*,* *Lepr-*mTagBFP2^+^CD45^−^Ter119^−^CD31^−^ stromal cells, LepR^+^ CD45^−^Ter119^−^CD31^−^ stromal cells. Data are representative of 6 experiments with one mouse per experiment.

**Supplementary Fig 6. Antibody staining penetration and specificity in optically cleared murine bone hemisections.** (**a**) Three-dimensional spatial images of a bone hemisection demonstrate the depth and structural integrity across its thickness, which exceeds 500µm. (**b**) Two-dimensional projected images of the bone hemisection indicated in panel **a**. A series of images (i-iii) are higher magnification images of the boxed area from panel **b**. Each slice depicted is 40µm thick at approximately 150µm intervals, representing different depths within the hemisection indicated in panel **a**. Each 40µm sections showed successful antibody staining of Lepr-mTagBFP2 (green) and endomucin (red), highlighting the clarity and resolution achievable with optical clearing techniques. (**c**) Assessments of antibody specificity through staining with different antibody combinations in optically cleared bone hemisections from 2-month-old *Scf^GFP/+^* mice. The middle section section shows the specificity of anti-GFP antibody staining specificity, while the lower section shows the specificity of endomucin antibody staining, ensuring that the observed fluorescence signals are not due to non-specific interactions. All images are representative of 3 experiments with one mouse per experiment.
